# A susceptible cluster in a religious camp allowed 2018–2019 measles outbreak in Japan: resurgence of a formerly eliminated virus

**DOI:** 10.3389/fpubh.2026.1813637

**Published:** 2026-06-08

**Authors:** Tetsuro Kobayashi, Hiroshi Nishiura

**Affiliations:** School of Public Health, Kyoto University, Kyoto, Japan

**Keywords:** mathematical model, *Paramyxoviridae*, statistical estimation, transmissibility, viral epidemic

## Abstract

**Objectives:**

We analyzed the 2018–2019 outbreak of measles in Japan, which started with a gathering at a religious youth camp in Mie, a prefecture located in the central part of Japan.

**Methods:**

A retrospective secondary analysis of cases from December 2018 to January 2019 was carried out. The index case was a Japanese man in his 20s living in Wakayama, a prefecture adjacent to Mie Prefecture. As part of his religious activities, he attended a youth camp in Mie Prefecture held from December 23 to 30, 2018. There were 28 identified secondary cases within the camp deriving from the index case, 2 additional household secondary transmissions from the index after the camp, and 31 additional reported cases from the attendees of the camp, for a total of 62 cases. Analyzing the publicly available data, including the first and last dates of exposure for each case, we calculated the incubation period, relative frequency of secondary transmission, estimated time of exposure during the camp, and effective reproduction number.

**Results:**

The incubation period had a mean of 11.2 days and variance of 5.3 days^2^. When the relative frequency of secondary transmission was assumed to follow a gamma distribution, the mean and variance were estimated to be 3.0 days and 0.3 days^2^, respectively. In the religious camp, the interquartile range (25th to 75th percentile) of infection time was 1.8 to 2.4 days prior to the onset of the primary case. The effective reproduction number during the religious camp was in the range of 30 to 32, which quickly dropped to <1 after the camp.

**Conclusion:**

We successfully show that a large outbreak of measles may happen in a cluster of unvaccinated individuals, especially in a closed environment such as a teenage camp. Strengthening routine immunization program by targeting unvaccinated populations would be vital.

## 1 Introduction

Measles is a highly contagious viral disease caused by the genus *Morbillivirus* in the *Paramyxoviridae* family. The disease is characterized by fever, maculopapular rashes, and catarrhal manifestations, usually following an incubation period of 10 to 14 days (ranging up to 23 days) after exposure (1). Because of its capacity for airborne transmission, the disease causes a large number of secondary transmissions per primary case (i.e., basic reproduction number), which ranges from 10 to 20 (2–4). While measles has yet to be eliminated in many parts of the world because of high transmissibility of the disease, vaccination failure, heterogeneous transmission patterns, and insufficient vaccination coverage, cases of clustered outbreaks within certain communities of people who refuse vaccination because of religious or tribal beliefs are often reported, even in countries where high vaccination coverage has been achieved (5–11).

In Japan, since the introduction of the measles-containing vaccine (MCV) in the mid-1960s, the prevalence of the local D5 strain of the virus has dramatically dropped, leading the nation to declare itself measles-free in 2015, and even afterwards, the routineimmunization coverage has been maintained above 90% (12–16). Nevertheless, multiple cases of local transmissions originating from an imported case have been reported. Aside from transmissibility, primary and secondary vaccination failure, vaccine refusal, and heterogeneous contact patterns over geographic space have been documented to play a role in allowing secondary transmission (17–20).

Before and after the COVID-19 pandemic, there have been measles outbreaks in highly vaccinated populations (21–23). The epidemic frequently happened in unvaccinated susceptible clusters (5), highlighting the need to look into vulnerable populations. The Western Pacific Region has not been exempted from the resurgence of measles outbreaks in the aftermath of disrupted vaccination program with COVID-19 pandemic. Even in a highly vaccinated population, the use of mathematical modelling techniques was shown to be useful, especially in evaluating the population level immunity (24, 36). Nevertheless, it has remained unclear how statistical models can assist the contact tracing practice, clarifying the epidemiological dynamics at the local level.

In the present study, we have analyzed the outbreak of measles that occurred in Japan in 2018–19, which started with a gathering at a religious youth camp held in Mie Prefecture, located in the central part of Japan. The exposure at the camp led to the infection of a large portion of the religious members who had refused vaccinations against any infections. Via retrospective assessment, the present study aimed to quantify the epidemiological dynamics of measles in an unprotected cluster of susceptible individuals, thereby objectively interpreting the course of outbreak.

## 2 Materials and methods

### 2.1 Epidemiological data

The present study is a retrospective cohort analysis employing mathematical models, carried out as a secondary analysis of cases from December 2018 to January 2019. We traced a dataset publicly released by the local government of Mie Prefecture. In Japan, it is mandatory that physicians report all diagnosed measles cases. The case definition of confirmed case was measles diagnosed by laboratory testing methods such as reverse-transcriptase polymerase chain reaction (PCR) or elevated IgM antibody levels using paired serum samples, or clinical measles cases exhibiting all three of the following clinical signs or symptoms: (i) fever, (ii) rash, and (iii) one of three inflammatory signs consisting of cough, coryza, and conjunctivitis) to the local health department in accordance with the Infectious Disease Law (25, 26). Undertaking contact tracing practice by local prefectures and the National Institute of Infectious Diseases, the local government published a “line-list” containing the date of onset, date of diagnosis, sex, age group (in 10-year increments), residential city and prefecture, and estimated place of infection of each case. In the outbreak considered here, the local governments of Mie and its surrounding prefectures collected the information on the vaccination status of each case.

### 2.2 Description of the outbreak

The index case was a Japanese man in his 20s living in Wakayama Prefecture, one of the neighboring prefectures of Mie Prefecture. This man belonged to a religious group that shares a belief that human spirits can heal themselves in case of injury or disease without taking any artificial (unnatural) substances such as vaccines or medications. As part of his religious activities, he attended a youth camp in Mie Prefecture held from December 23 to 30, 2018. He had a fever starting on December 28, the 4th day of the youth camp, followed by skin rash that appeared on December 30, the last day of the camp. Because he had no recent travel history, the route of transmission is unknown. Excluding the index case, 53 members of the religious group, mainly school children and adults up to age 40 from various places in Japan, attended the camp.

**Figure 1:**
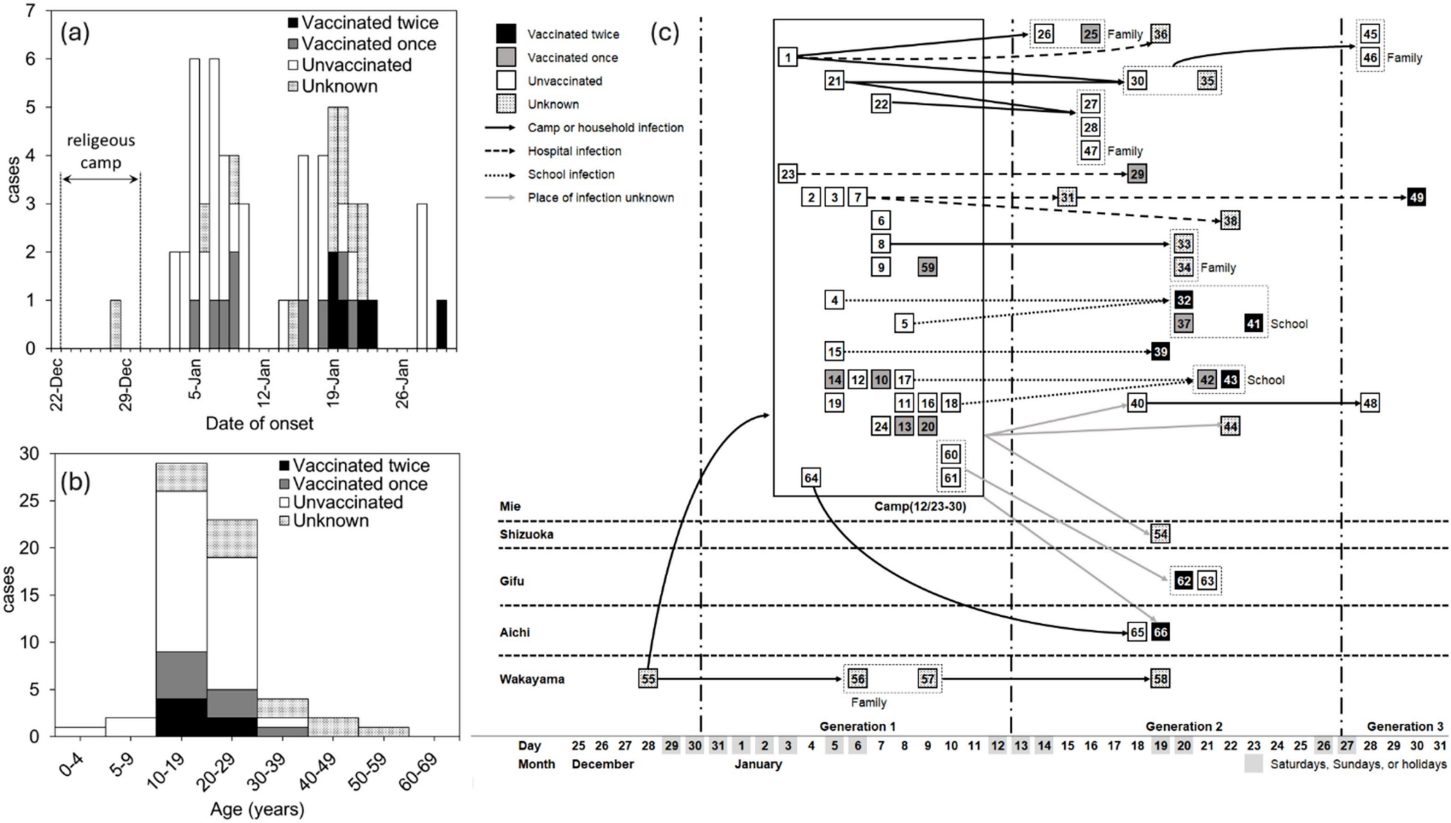
Epidemiological profile of measles outbreak in Mie prefecture, 2018–19. **(a)** Daily incidence count stratified by vaccine status. **(b)** Age distribution of cases stratified by vaccine status. **(c)** Transmission tree of measles outbreak in Mie prefecture, 2018–19. Each individual, indicated by an icon with a unique number, is aligned according to the date of onset. Vaccination status is indicated by icon’s grey density.

There were 28 secondary cases within the camp transmitted from the index case and 2 additional household secondary transmissions from the index after the camp. Thirty-one additional secondary transmissions from the attendees of the camp were later reported. Thus, there was a total of 62 cases in the outbreak, of which 29 involved teenagers (Figure 1a). Of the 50 cases with known vaccination status, 29 involved attendees of the camp. The vaccination status of the 25 attendees of the camp who evaded infection is unknown. The profiles of all cases in the present outbreak are summarized in Supplementary Table 1. Because the transmission network data that show who acquired an infection from whom was available, we were granted an opportunity to visualize the transmission tree shown in Figure 1c. There were 30 first-generation secondary transmissions by the index case, of which 20 affected teenagers and 23 affected unvaccinated people. The second generation consisted of 27 persons, 9 of whom were teenagers and 10 of whom were unvaccinated. The third generation consisted of 4 persons, 3 of whom were unvaccinated.

(1) Incubation period estimation

We first estimated the incubation period *f*(*τ*) assuming that the probability density function for the time *τ* follows a lognormal distribution with mean *μ* and standard deviation *σ*. We used datasets of the onset date of symptoms in all secondary cases, both inside and outside of the religious camp. Each infected person, including the index case, was assigned a unique integer *i* ∈ [1, 62]. From the publicly available data, the possible calendar dates during which each secondary case *i* was exposed to the infected primary case were identified, with the first and last days of exposure defined as *E*_L,i_ and *E*_R,i_, respectively. Then, addressing the censored exposure data, we used the following likelihood function Equation 1 to estimate parameters:


$$\begin{array}{c} L(\Theta )=\prod_{i}\frac{1}{E_{R,i}-E_{L,i}}\int_{E_{L,i}}^{E_{R,i}}f(x_{i}-e)\mathrm{de} \end{array}$$ (1)

Θ∈(μ,σ)

Here, *x*_i_ is the date of symptom onset in secondary case *i*. We then compared the estimated incubation period with those assuming other distributions calculated in published studies (27, 28). To calculate the 95% confidence interval (95% CI), we performed bootstrap resampling with replacement 1,000 times using the same number of cases.

(2) Relative frequency of secondary transmission

Secondary transmission of measles occurs well before the onset of the primary case (29). The relative infectiousness of a secondary case *i u* days before or after the onset of primary case *λ*_i_(*u*) is estimated using the Equation 2 shown below (30):


$$E[N(\mathrm{\Delta t})]=\sum_{i\in [1,61]}\int_{u_{\min}}^{\mathrm{\Delta t}}\lambda_{i}(u)f(\mathrm{\Delta t}-u)\mathrm{du}$$ (2)

Assuming that the serial interval count is sufficiently captured by a Poisson distribution, we have


$$-\ln L(\Theta )=\sum_{\mathrm{\Delta t}}(E[N(\mathrm{\Delta t})]-N(\mathrm{\Delta t})\cdot \ln(E[N(\mathrm{\Delta t})]))$$

$$\Theta \in (\theta ,k)\;\text{or}\;\lambda_{u}\;(u=[-4,5])$$

Here, *N*(Δ*t*) is the observed number of serial intervals of length Δ*t*, i.e., days between the onsets of the primary and secondary cases. We assumed that the relative infectiousness *λ*_i_(*u*) follows a gamma distribution of shape and scale parameters *θ* and *k*, respectively, or step function model *λ*_i,u_ every day. We did not use the incubation period calculated in the subsection (1), but instead we assume that *f*(.) was known and followed a lognormal distribution, as calculated in published studies (27, 28). Hereafter, estimate from historical data refers to the household transmission data (27), while that from a systematic review refers to the one published in 2009 (28). We performed resampling with replacement 1,000 times using the same number of cases to calculate the 95% confidence interval (95% CI).

(3) Estimated time of exposure in the camp

Using an alternative approach, we analyzed the most likely time of the superspreading event involving 28 persons infected from the index case at the religious camp. Considering the in-camp outbreak as a point-source outbreak, we estimated the most likely time of exposure during the camp from December 23 to 30, 2018. As mentioned in subsection (2), we assumed that the incubation period was known and followed a lognormal distribution, with its parameters fixed at the values estimated in published studies (27, 28, 31). The date of secondary transmission by the index case, denoted by *γ*, was estimated by calculating the likelihood, as shown in Equation 3, that the length of incubation was *x*_i_ – *γ* days in secondary case *i*, with x_i_ denoting the observed calendar date of the onset of case *i*:


$$L_{i}(\gamma ;x_{i},\mu ,\sigma )=\frac{1}{(x_{i}-\gamma )\sigma \sqrt{2\pi }}\exp(-\frac{{\left(\ln(x-\gamma )-\mu \right)}^{2}}{2\sigma^{2}}),$$ (3)

yielding the negative log likelihood


$$\mathrm{NLL}=-\sum_{i}\ln L_{i}(\gamma ;x_{i},\mu ,\sigma )$$

We performed resampling with replacement 1,000 times using the same number of cases to draw a boxplot indicating the distribution at the following percentiles: 97.5, 75, 50, 25, and 2.5%.

(4) Effective reproduction number

The incidence of symptom onsets as a function of calendar time *t*, denoted by *c*(*t*), can be backcalculated by taking the integral of the incidence of infection occurring *τ* days earlier multiplied by the incubation period *f*(*τ*), as shown in Equation 4:


$$\begin{array}{c} E[c(t)]=\int_{0}^{\infty }i(t-\tau )f(\tau )\mathrm{d\tau} \end{array}$$ (4)

where *i*(*t*) is the incidence of infection. Because the event of infection is unobservable, we use the fact that the secondary transmission occurring at calendar time *t* is the integral of the onset of primary cases *u* days earlier multiplied by the relative infectiousness of the primary case *λ*(*u*):


$$\begin{array}{c} i(t)=R(t)\int_{-u_{\min}}^{\infty }c(t-u)\lambda (u)\mathrm{du} \end{array}$$ (5)

where $u_{\min}$ is the minimum possible number of days with infectiousness dating back from the illness onset date. Here, *R*(*t*) is the effective reproduction number at calendar time *t*. Plugging Equation 5 into Equation 4, we obtain the expected number of onsets at time *t* using observable onset data:


$$\begin{array}{c} E[c(t)]=\int_{0}^{\infty }R(t-\tau )f(\tau )\int_{-u_{\min}}^{\infty }c(t-\tau -x)\lambda (x)\text{dxdτ} \end{array}$$ (6)

Because we only had daily data of symptom onsets, we used a discrete form:


$$E[c_{t}]=\sum_{\tau =0}^{\infty }R_{t-\tau }f_{\tau }\sum_{u=u_{\min}}^{\infty }c_{t-\tau -u}\lambda_{u}$$

We assumed that the relative infectiousness *λ*_u_ follows a step-function model varying every 5 days. Again, the incubation period was assumed as known and to follow a lognormal distribution, according to published data (27, 28). We then fit the expected number of symptom onsets to the observed data, assuming Poisson-distributed variations, minimizing the negative logarithm:


$$-\ln L(\Theta )=\sum_{t}(E[c(t)]-c(t)\cdot \ln(E[c(t)]))$$

Upon estimation, we calculated the inverse of the Hessian matrix and used multivariate normal distributions to perform bootstrap resampling of parameters 1,000 times to calculate the 95% CI. All statistical analyses were performed using R version 4.4.1 (The R Project for Statistical Computing, Vienna, Austria).

### 2.3 Ethical considerations

The present study used only publicly available secondary data, without any identifiable information.

## 3 Results

**Figure 2:**
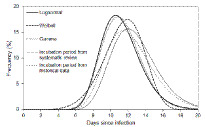
Frequency of the incubation period of measles as a function of days since infection. Frequency of the incubation period of measles in days since infection assuming a lognormal distribution, compared with the incubation period distribution estimated in a systematic review (28) and historical data (27).

Using model (1), the incubation period assuming a lognormal distribution had a mean of 11.2 days and a variance of 5.3 days^2^. The incubation periods estimated in a systematic review and in historical data, as shown in Figure 2, consistently have a mean of 12.8 days and 12.3 days, respectively.

**Figure 3:**
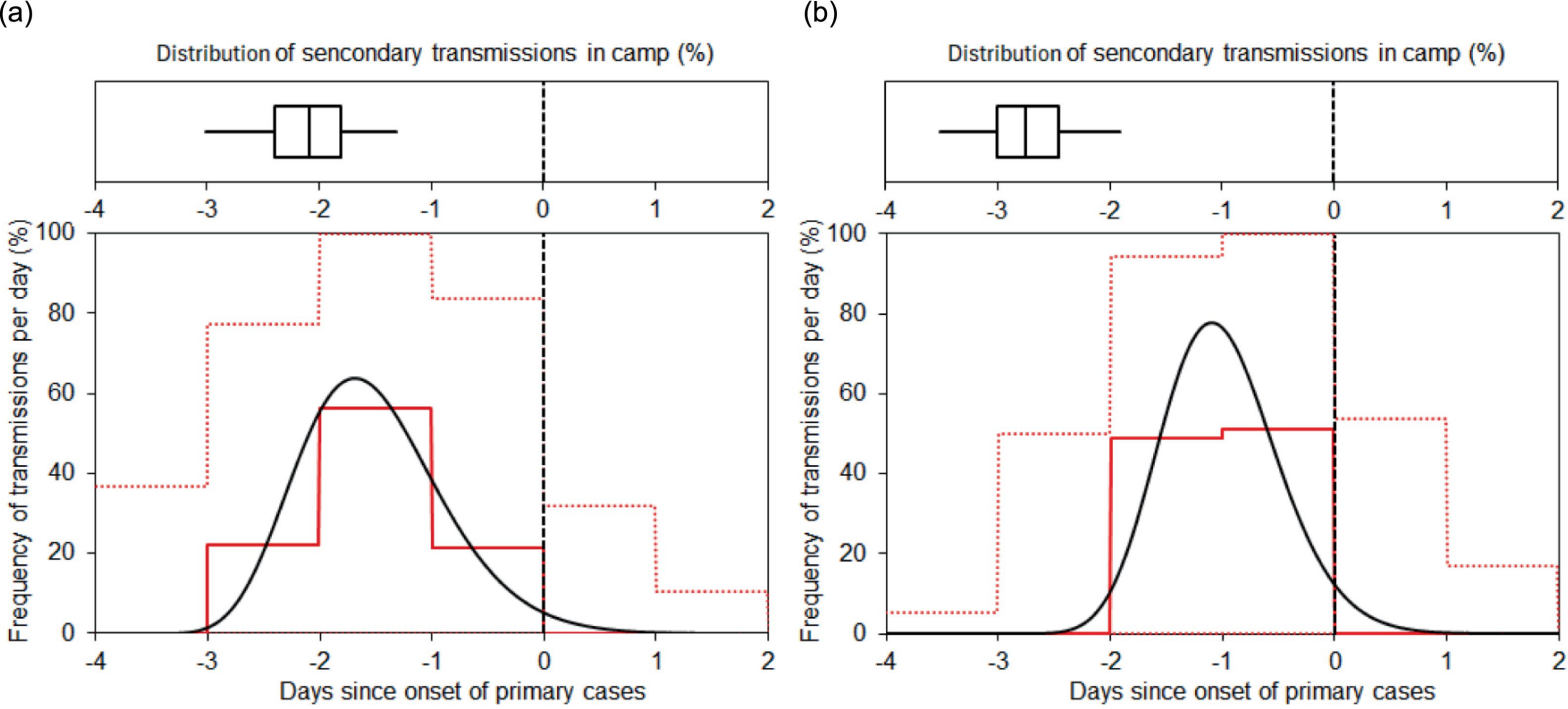
Boxplot distribution of secondary transmissions in camp compared with relative frequency of secondary infections in all cases. The relative frequency of secondary infections is assumed to follow a gamma distribution (in black) or step function (in red) as a function of days since the onset of primary infection. Incubation period was based on **(a)** a systematic review (28) and **(b)** historical data (27).

The relative frequency of secondary transmission is shown in Figure 3. When the incubation periods were fixed at the values estimated from historical data, the frequency peaked at 0 to 1 days prior to the onset of the primary case. When the relative frequency was assumed to follow a gamma distribution, the mean and variance were 3.0 days and 0.3 days^2^, respectively. With respect to the estimated time of exposure during the religious camp, the interquartile range (25th to 75th percentile) was 1.8 to 2.4 days prior to the onset of the primary case. When the incubation period was fixed at the values provided in a systematic review, the estimated time peaked 1 to 2 days prior to the onset of the primary case. When the time of exposure was assumed to follow a gamma distribution, the mean and variance were 2.5 days and 0.4 days^2^, respectively.

**Figure 4:**
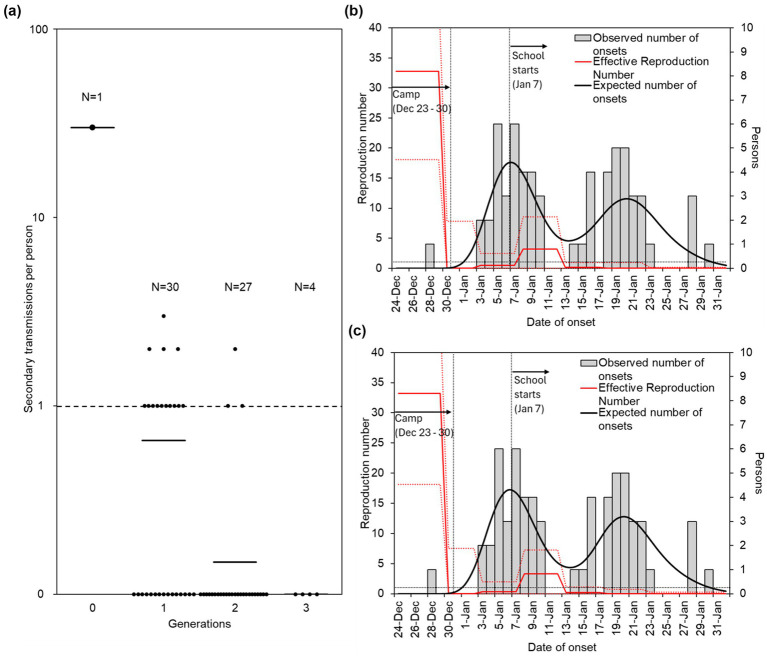
Time-dependent and generation-dependent reproduction number. **(a)** Generation-dependent distribution of secondary infection per primary case. Mean number of secondary infections in each generation is marked by a solid horizontal line. **(b,c)** Time-dependent reproduction number superimposed with observed and estimated number of cases as a function of the date of illness onset. For the incubation period, we referred to a systematic review (28) for **(b)** and historical data (27) for **(c)**.

Figure 4a shows the number of secondary transmissions in each generation. The average corresponds to the generation-dependent effective reproduction number. The effective reproduction number superimposed on the epidemic curve with respect to onset date is shown in Figures 4b,c, in which the incubation period is fixed at values estimated from either historical data or a systematic review, respectively. In both cases, the effective reproduction number quickly dropped below the threshold value of 1 when the religious camp ended on December 30. It slightly surpassed the value of 1 for 5 days when school started on January 7.

### 3.1 Discussion

In the present study, we have estimated the incubation period and relative frequency of secondary transmission and quantified the transmission dynamics of the 2018–2019 measles outbreak in Mie, Japan. The incubation period was comparable with values from historical data (27, 32) and a systematic review (28), except that the mean was approximately 1 day shorter in our analysis. The relative frequency of secondary transmission was the highest 1 or 2 days before the onset of fever in the primary case. The effective reproduction number during the religious camp was on the order of 30, comparable with the conventional values of basic reproduction number, 10 to 20 in a fully susceptible population (4, 24, 33–36). Of note, 23 of 28 secondary transmissions that occurred in the camp involved unvaccinated persons, while the remaining 5 involved persons only vaccinated once. However, the effective reproduction number quickly dropped to <1 after the camp. This leads us to objectively conclude that the outbreak in the camp offered an opportunity for a superspreading event to begin because of an extraordinarily low level of vaccination coverage within the attendees’ community.

What can we learn from the fact that a superspreading event involving 28 secondary infections in a closed environment was observed in a religious youth camp? The religion that ran the camp inspired followers not to receive vaccinations, and as a result, the secondary transmissions during the camp occurred mostly in unvaccinated individuals. The attendees, including the index case, were mostly teens coming from all over Japan and did not know each other’s vaccination statuses before the camp. Critically, the camp possibly led to the clustering of a susceptible population at high risk of measles, and in such an instance, a single infectious case in the group acted as a dynamic avenue to cause a large number of secondary transmissions. Depending on the models used, the effective reproduction number was in the range of 30 to 32 during the camp, while it swiftly dropped to below 1.0 after the camp. The effective reproduction number on the order of 30 even exceeded published and frequently quoted estimates from 10 to 20. We consider it to have been caused by clustering effect in close contact setting of susceptible individuals, but it could also be possibly due to random error caused by limited sample size. Although the effective reproduction number slightly increased to 3.2 to 3.3 again when school reopened on January 7, it remained below 1 until the outbreak ended, probably because of high vaccination coverage in the community, where coverage was >90% (37).

Even though the herd immunity threshold of measles given the basic reproduction number from 10 to 20 is 90—95%, it must be remembered that susceptible individuals are often clustered. As we described in Introduction, there have been well documented studies of measles in a cluster of susceptibles individuals across the world (5, 6), which tended to be greater in the size than what we presented. What our study methodologically adds to literature is that our approaches can greatly help interpreting the transmission dynamics during the localized outbreak. Estimating the incubation period, it enabled ascertaining the time window of exposure via multiple approaches (Figure 3), and we were able to objectively show that the exposure most likely happened during the religious camp in Mie. Moreover, not only reconstructing the transmission tree (Figure 1) but also we computed the effective reproduction number. Both the tree and time-dependent estimate of the reproduction number allowed us to objectively interpret the local epidemic trajectory, again via multiple approaches (Figure 4). Statistical modelling can greatly assist the contact tracing practice.

In religious communities, shared beliefs can lead to the clustering of susceptible individuals, increasing the risk of superspreading outbreaks. In the outbreak considered here, this occurred as a result of a dynamic change in the population, with susceptible individuals gathering from other distant places where herd immunity had been achieved. In fact, there have been several reports of measles outbreaks occurring in specific communities that share an ideology and remain unvaccinated (5–9, 11, 21–23, 38, 39). Thus, achieving uniformly high vaccination coverage across all subpopulations remains difficult, even in places such as North America with sufficient vaccination coverage against the pathogen. Such clustering is not limited to religious groups; it may also occur among migrants, indigenous communities, and other vaccine-hesitant communities, or others who share common beliefs or behaviors. Therefore, assessing the degree of susceptibility of clustering across diverse groups before an outbreak occurs is essential for preparedness. This is we believe an important caveat of this study: rather than focusing our effort on response to outbreak events, it is vital to identify susceptible clusters in advance of the exposure. Particular attention must also be paid to the mobility of susceptible individuals, as moving clusters can amplify the difficulty of containment efforts (e.g., contact tracing at multiple distant geographic locations).

The superspreading event was restricted to the religious youth camp, where unvaccinated susceptible individuals gathered. After the infected attendees dispersed to their respective home cities, where relatively high vaccination coverage has been achieved, no further superspreading events were observed. If such susceptible individuals had come into contact with other clusters of susceptibles after the camp, larger outbreaks might have been observed in many other places. In the case of Mie outbreak, the superspreading event remained confined to a cluster of unvaccinated individuals, while transmission did not spread widely in the general population, perhaps due to high vaccination coverage achieved in Japan (12–16, 33). This highlights the critical role of vaccination in preventing broader community transmission, even when localized clusters of susceptible individuals may exist. Moreover, this outbreak began in the urban region of Mie prefecture, but contact tracing efforts are typically constrained by local governments. Given that the seminar involved travel across prefectures, timely active surveillance proved challenging. The mobility of the susceptible population further complicated outbreak control efforts. It is important to realize that broader community transmission can be prevented if those outside such susceptible clusters are sufficiently immunized. In other words, localized outbreaks can be prevented from growing into community-wide epidemics if surrounding herd immunity is secured.

There are several limitations of our study. First, the small sample size may not accurately capture the transmission dynamics. For instance, the incubation period distribution in our study is slightly shorter than those found in references (27, 28). This discrepancy may also lead to overestimation of peak infectiousness, i.e., 2 to 3 days before the onset of illness. Second, the level of heterogeneity within the camp was unknown. In particular, the vaccine status and age distribution of those who attended the camp but evaded infection remain unknown. Such heterogeneity should be considered for better description of the outbreak. Third, we do not see a direct causality between public announcements and low reproduction number in the later phase of the outbreak after January 11. Fourth, because we only have vaccination status for infected individuals during the camp, we were unable to explicitly assess the vaccine effectiveness of the local population. If the majority of people who escaped from infection were vaccinated, this could introduce a selection bias and affect the estimation of the susceptibility of the camping cluster and thus the reproduction number. Finally, because the public data outside of Mie Prefecture partly did not clarify the connection between the cases reported and the exposure in the camp, the number of secondary infections within the camp may be underestimated. Mie Prefecture reported that out of the 54 persons who attended the camp, 38 were Mie residents and 16 were residents of other prefectures; 29, including the index case, and 4 of these persons were reported as “camp-related,” respectively. Thus, “camp-related” individuals accounted for 74 and 25% of the attendees from inside and outside of the prefecture, respectively. As shown in the supplementary table, between January 4 and 12, Osaka reported 6 other cases of measles in teens and young adults in their 20s and 30s who were either unvaccinated or vaccinated once, although whether they had attended the camp before the onset is unknown. If these six people were all attendees of the camp, the reproduction number in the camp would have been as high as 38, instead of 32 (Supplementary Table 2).

## 4 Conclusion

We successfully showed that a large outbreak of measles may happen in a cluster of unvaccinated individuals, especially in a closed environment such as a teenage camp. The infectiousness of measles was calculated to be 2 to 3 days before the onset of illness. The effective reproduction number quickly fell below 1 when the camp ended and public announcements were released. Strengthening routine immunization program by targeting unvaccinated populations would be vital.

## Data Availability

The original contributions presented in the study are included in the article/Supplementary material, further inquiries can be directed to the corresponding author.
